# Accuracy and Agreement of a 45 mg Versus a 75 mg ^13^C-Urea Breath Test for the Diagnosis of *Helicobacter pylori* Infection in Adults: A Randomized Crossover Trial

**DOI:** 10.3390/diagnostics16101567

**Published:** 2026-05-21

**Authors:** Jun Huang, Yaohong Xie, Jian Huang, Bingyun Lu, Ye Chen

**Affiliations:** 1 Guangdong Provincial Key Laboratory of Gastroenterology, Department of Gastroenterology, Nanfang Hospital, Southern Medical University, Guangzhou 510000, China; wlncsft@smu.edu.cn (J.H.);; 2 Integrative Microecology Clinical Center, Shenzhen Key Laboratory of Gastrointestinal Microbiota and Disease, Shenzhen Clinical Research Center for Digestive Disease, Shenzhen Technology Research Center of Gut Microbiota Transplantation, Department of Gastroenterology, Shenzhen Hospital, Southern Medical University, Shenzhen 518000, China

**Keywords:** *Helicobacter pylori*, ^13^C-urea breath test, randomized crossover trial, diagnostic agreement, diagnostic accuracy

## Abstract

**Background:** The optimal ^13^C-urea breath test (UBT) dosage for diagnosing *Helicobacter pylori* remains debated. We compared the accuracy and agreement of a low-dose 45 mg tablet (without citric acid) versus the standard 75 mg granule (with citric acid) protocol. **Methods:** In this prospective, randomized crossover trial, adults underwent both a 45 mg and a 75 mg ^13^C-UBT on the same day. Breath samples were collected at 15 and 30 min. The primary outcome was diagnostic agreement at 30 min. Secondary outcomes included diagnostic performance against a composite reference standard, wherein concordant 45 mg and 75 mg UBT results were presumed to represent true *H. pylori* status, while discordant cases underwent endoscopic reference testing (rapid urease test and histology, with immunohistochemistry where required), stratified by age and BMI. **Results:** For the 431 participants included, the 45 mg and 75 mg tests showed substantial agreement at 30 min (90.3%; κ = 0.766). The 30 min sampling time yielded significantly better accuracy than 15 min for both doses. The 45 mg protocol achieved excellent accuracy (AUC 0.953), statistically non-inferior to the 75 mg protocol (AUC 0.966; *p* = 0.50). Notably, in participants aged <40 years or with BMI < 25.0 kg/m^2^, the 45 mg protocol demonstrated robust performance (AUC 0.977 and 0.966, respectively), comparable to the 75 mg standard (*p* > 0.05). No adverse events occurred. **Conclusions:** The low-dose 45 mg ^13^C-UBT provides high diagnostic agreement and comparable clinical performance to the standard 75 mg protocol without requiring citric acid acidification. Its robust performance in younger, lower-BMI individuals shows clinical promise. However, because diagnostic accuracy analyses partly relied on a composite reference assumption, further externally validated studies are required before broad screening-policy claims can be made.

## 1. Introduction

*Helicobacter pylori (H. pylori)* infection represents a significant global public health challenge, with a prevalence exceeding 50% worldwide and reaching as high as 70–90% in developing nations [1,2]. This spiral-shaped, Gram-negative bacterium colonizes the human gastric mucosa and is a primary etiological agent for various gastrointestinal diseases, including chronic gastritis, peptic ulcer disease, gastric mucosa-associated lymphoid tissue (MALT) lymphoma, and gastric adenocarcinoma [3]. Recognizing its carcinogenic potential, the International Agency for Research on Cancer (IARC) of the World Health Organization classified *H. pylori* as a Class I carcinogen in 1994 [4,5]. Consequently, the accurate and timely diagnosis of *H. pylori* is crucial for effective clinical management, eradication therapy, and the prevention of long-term complications [6,7].

Among the available diagnostic modalities, the ^13^C-Urea Breath Test (^13^C-UBT) has emerged as the leading non-invasive method [3,8]. Owing to its high accuracy, specificity, and convenience, the ^13^C-UBT is widely recommended by major international and national consensus guidelines—including the Kyoto Global Consensus, Maastricht V/VI Consensus, and American College of Gastroenterology (ACG) guidelines—as a preferred choice for both initial diagnosis and confirmation of eradication post-treatment [9,10,11,12]. This test relies on the principle that *H. pylori*’s urease enzyme hydrolyzes orally administered ^13^C-labeled urea, releasing ^13^C-labeled carbon dioxide (^13^CO_2_) that can be detected in the patient’s exhaled breath [13].

Despite its widespread clinical application, the optimal dosage of ^13^C-urea remains a subject of considerable debate. Historically, doses have progressively decreased from as high as 350 mg, with current clinical practice commonly employing either a 75 mg or a 45 mg dose [14]. While some practitioners believe that a higher dose (75 mg) may yield a stronger signal and thus greater accuracy, emerging evidence suggests that lower doses may be equally effective [15,16]. In fact, several studies have demonstrated that ^13^C-UBT doses as low as 15–50 mg can achieve diagnostic sensitivity and specificity exceeding 95% [17,18,19]. Lowering the dose offers significant clinical and economic advantages. Specifically, reducing the dosage directly lowers test costs, a critical factor for large-scale population screening programs and in resource-limited settings. Additionally, it minimizes patient exposure to stable isotopes, aligning with the principles of diagnostic stewardship. From a workflow perspective, demonstrating the efficacy of a lower-dose tablet could simplify supply chain logistics compared to bulkier granule formulations. While lower doses may potentially enhance specificity by minimizing false-positive results from oral urease-producing bacteria, the influence of the drug formulation—such as tablets versus granules—on dissolution, gastric distribution, and ultimately, diagnostic performance has not been fully elucidated [20].

Therefore, clarifying the non-inferiority of the 45 mg dose has substantial policy implications for updating testing guidelines. To address this knowledge gap, we designed this prospective, randomized, crossover trial. The primary objective of our study was to directly compare the diagnostic accuracy and agreement between a low-dose 45 mg ^13^C-urea tablet and a standard-dose 75 mg ^13^C-urea granule formulation for the diagnosis of *H. pylori* infection in a cohort of adult participants.

## 2. Methods

### 2.1. Trial Design and Oversight

We conducted a prospective, single-center, randomized, open-label, two-period crossover trial at Shenzhen Hospital, Southern Medical University, in Shenzhen, China [21]. The trial was designed to evaluate the diagnostic agreement between a low-dose (45 mg) and a standard-dose (75 mg) ^13^C-urea breath test (UBT). In this design, each participant served as their own control.

The study was conducted in accordance with the principles of the Declaration of Helsinki and Good Clinical Practice guidelines [22]. The protocol (version 1.2) was approved by the Medical Ethics Committee of Shenzhen Hospital, Southern Medical University (approval no. NYSZYYEC20230016). All participants provided written informed consent before enrollment. The trial was prospectively registered at the Chinese Clinical Trial Registry (ChiCTR2400090596; registration date: 9 October 2024). Patients and the public were not involved in the design, conduct, or reporting of this study. The funders had no role in the study design; in the collection, analysis, or interpretation of the data; or in the decision to submit the manuscript for publication. The authors vouch for the accuracy and completeness of the data and for the fidelity of the trial to the protocol.

### 2.2. Participants

We enrolled participants from October 2023 through June 2024 from the gastroenterology outpatient clinic. Eligible participants were adults between 18 and 75 years of age scheduled to undergo a ^13^C-UBT for the diagnosis of *H. pylori* infection.

Exclusion criteria included the use of proton-pump inhibitors or H_2_-receptor antagonists within the previous 2 weeks or the use of antibiotics or bismuth-containing compounds within the previous 4 weeks, in line with current clinical guidelines [10,23]. Additional criteria were a history of *H. pylori* eradication therapy within 4 weeks, participation in another interventional clinical trial, severe comorbidities that would preclude completion of the procedures, and pregnancy or breastfeeding.

### 2.3. Randomization and Blinding

Participants were randomly assigned in a 1:1 ratio to one of two sequences: the 45 mg test followed by the 75 mg test (Sequence A), or the 75 mg test followed by the 45 mg test (Sequence B). The randomization sequence was generated with the use of SAS software (version 9.4, SAS Institute Inc., Cary, NC, USA) with permuted blocks of eight by a statistician who was not involved in patient enrollment. The allocation sequence was concealed in a secure, central, password-protected database and was revealed sequentially to the clinical research coordinator only after a participant had been confirmed as eligible and had provided consent.

The trial was open-label because of the explicitly labeled dosages on the commercial test kits. However, personnel analyzing the automated breath-sample results, pathologists evaluating histologic specimens, and the data analysts were unaware of the sequence assignments until the analysis was complete.

### 2.4. Study Procedures

All procedures were performed in the morning after an overnight fast. Participants underwent both the 45 mg and 75 mg ^13^C-UBT interventions on the same day, separated by a washout period of at least two hours. For each intervention, a baseline (0 min) breath sample was collected before the participant ingested the assigned dose: either a 45 mg tablet (without citric acid) or 75 mg of granules (containing citric acid) dissolved in water. Post-dose breath samples were collected at 15 and 30 min. All breath samples were analyzed for ^13^CO_2_ enrichment with the use of a single infrared spectrometer (Breath ID, Exalenz Bioscience Ltd., Modiin, Israel) [24].

For participants with discordant results between the two ^13^C-UBT interventions, a definitive diagnostic classification was determined by an endoscopic reference standard. *H. pylori* infection status was defined based on the concordance of the rapid urease test (RUT) and histology [25]. Participants were classified as *H. pylori*-positive if both the RUT and histology were positive, or if histology alone was positive (given its higher specificity). Conversely, participants were classified as negative only if both tests yielded negative results. Rapid urease testing and histology were performed for all patients undergoing endoscopy. In cases of discordance where RUT was positive but routine histology was negative, additional immunohistochemical staining was performed to confirm the presence of *H. pylori*; if immunohistochemistry was negative, the patient was classified as uninfected.

### 2.5. Outcomes

The pre-specified primary outcome was the diagnostic agreement between the 45 mg and 75 mg ^13^C-UBTs. Agreement was selected as the primary endpoint because performing invasive endoscopy (the reference standard) on all participants, particularly asymptomatic individuals in a screening context, was considered ethically unjustified and logistically challenging. Therefore, the study was powered to primarily demonstrate that the 45 mg protocol yields results that are statistically concordant with the standard 75 mg clinical protocol.

Pre-specified secondary outcomes included the diagnostic performance (sensitivity, specificity, predictive values, and area under the receiver operating characteristic curve [AUC]) of four test protocols (45 mg and 75 mg doses, each with 15 and 30 min samples). Additional secondary outcomes were subgroup analyses of the primary outcome and an analysis of discordant cases against the endoscopic reference standard [26,27]. For the diagnostic accuracy analyses (sensitivity, specificity, and AUC) of the full per-protocol population, a composite reference standard was applied: participants with concordant results between the 45 mg and 75 mg tests were presumed to represent their true *H. pylori* status, while the definitive *H. pylori* status for those with discordant results was determined by the endoscopic reference standard. The safety outcome was the incidence of any adverse events, systematically collected via a telephone follow-up one week after the procedure.

### 2.6. Statistical Analysis

The sample size was calculated to provide 80% power for a non-inferiority analysis of the sensitivity and specificity of the 45 mg test as compared with the 75 mg test, with a non-inferiority margin of −10 percentage points and a one-sided alpha of 0.025 [28]. All other statistical analyses were pre-specified and performed with the use of SPSS software (version 26.0, IBM Corp., Armonk, NY, USA). A two-sided *p* value of less than 0.05 was considered to indicate statistical significance.

The primary efficacy analysis was conducted on all randomized participants who had an evaluable result for both ^13^C-UBT interventions (the per-protocol population). We assessed diagnostic agreement by calculating the overall percent agreement and Cohen’s kappa coefficient, each with their corresponding 95% confidence intervals (CIs). The McNemar test was used to assess systematic disagreement. For secondary outcomes, we calculated sensitivity, specificity, and predictive values with 95% CIs. We compared AUCs with the use of the method of DeLong et al. [29]. Pre-specified subgroup analyses were conducted to assess the consistency of the primary outcome. Age was selected as a stratification factor because aging is associated with an increased prevalence of atrophic gastritis and intestinal metaplasia, which can reduce urease activity and potentially affect UBT sensitivity [30,31]. The cutoff of 40 years was chosen based on epidemiological data from East Asian populations showing a significant increase in the prevalence of atrophic gastritis and intestinal metaplasia in individuals aged 40 years and older compared to those under 40 [32]. All analyses were performed on a complete-case basis; no imputation was used for missing data.

## 3. Results

### 3.1. Participant Flow and Baseline Characteristics

**Figure 1:**
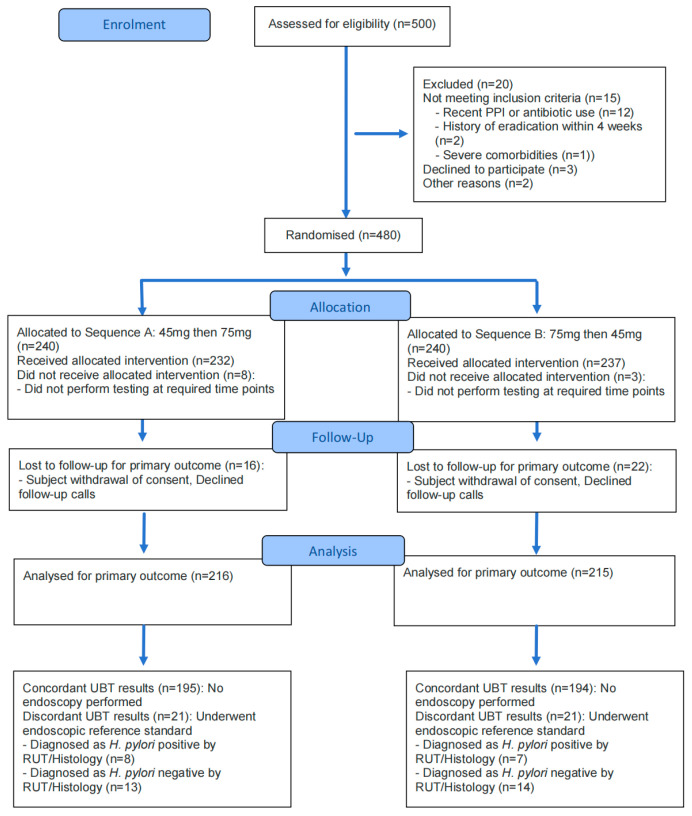
Enrollment, randomization, and follow-up of trial participants. Of the 500 participants assessed for eligibility, 480 underwent randomization to one of two crossover sequences. A total of 431 participants completed both study interventions and were included in the per-protocol analysis for the primary outcome. To establish the composite reference standard for secondary diagnostic accuracy analyses, participants with concordant 30 min UBT results (*n* = 389) were presumed to reflect their true *H. pylori* status without invasive testing. The 42 participants with discordant UBT results underwent the definitive endoscopic reference standard (RUT and histology) to determine their final infection status.

From October 2023 through June 2024, a total of 500 participants were assessed for eligibility. Of these, 20 were excluded (15 did not meet inclusion criteria primarily due to recent proton-pump inhibitor or antibiotic use, 3 declined to participate, and 2 were excluded for other reasons), and 480 underwent randomization to one of two crossover sequences. A total of 431 participants completed both study interventions and were included in the per-protocol analysis for the primary outcome (Figure 1, File S1).

The two randomized sequence groups were well balanced with respect to all pre-specified baseline demographic and clinical characteristics (Table 1). The mean age of the participants was 42.1 years, and 255 (58.9%) were female. Regarding the clinical indication for testing, the majority of participants (336 [78.0%]) underwent the UBT for initial diagnosis, while 95 (22.0%) were tested for post-eradication confirmation. There were no significant differences between the groups in any of the baseline variables.

### 3.2. Intervention Delivery

Of the 480 randomized participants, 469 (97.7%) received both the 45 mg and 75 mg ^13^C-UBT interventions as allocated in their assigned crossover sequence. Protocol deviations related to the administration of the interventions were recorded in 11 participants (2.3%). In Sequence A (45 mg → 75 mg), 8 of 240 participants (3.3%) did not receive the interventions as planned; in Sequence B (75 mg → 45 mg), 3 of 240 participants (1.3%) did not. These deviations were due to the participants’ inability to provide breath samples at the pre-specified 15 and 30 min intervals. Adherence to the single-dose administration for each period was otherwise complete. The actual mean time interval between the two ^13^C-UBT interventions was 153.17 min (153.19 min for Sequence A and 153.15 min for Sequence B). This interval of over 2.5 h was implemented to minimize potential carryover effects from the initial isotope administration. Furthermore, consistent with our rigorous exclusion criteria, no concomitant medications known to interfere with ^13^C-UBT results (such as proton-pump inhibitors, H2-receptor antagonists, bismuth, or antibiotics) were reported during the testing period.

### 3.3. Outcomes

#### 3.3.1. Primary Outcome: Diagnostic Agreement

In the per-protocol population of 431 participants, the primary outcome was the diagnostic agreement between the 45 mg and 75 mg tests at 30 min. The overall agreement was 90.3%, with a Cohen’s kappa coefficient of 0.766 (95% CI, 0.698 to 0.834), indicating substantial agreement (Table 2). The McNemar test showed no significant systematic disagreement between the two tests (*p* = 0.088). Agreement was consistent when analyzed by randomization sequence. At the 15 min time point, the overall agreement was 87.0% (κ = 0.686).

#### 3.3.2. Secondary Outcomes: Diagnostic Performance

**Figure 2:**
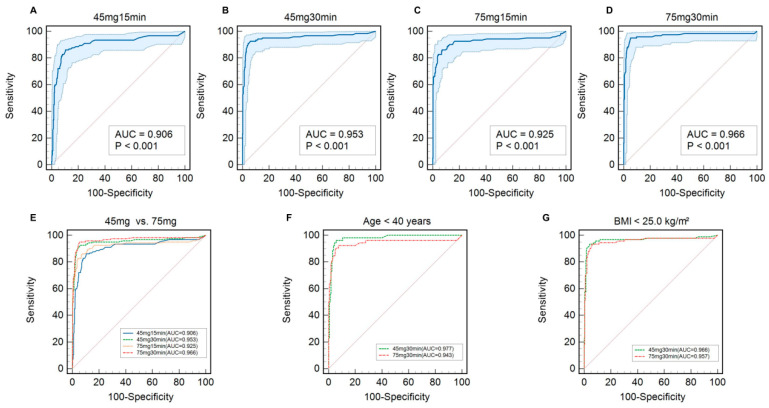
Diagnostic performance of four ^13^C-urea breath test protocols overall and in key subgroups. Shown are the receiver operating characteristic (ROC) curves for the diagnostic performance of the four test protocols. The performance in the total per-protocol population (*n* = 431) is shown for the 45 mg, 15 min test (Panel **A**); the 45 mg, 30 min test (Panel **B**); the 75 mg, 15 min test (Panel **C**); and the 75 mg, 30 min test (Panel **D**). A composite overlay of these four protocols is shown in (Panel **E**). Head-to-head comparisons of the two 30 min protocols are shown for participants younger than 40 years of age (Panel **F**) and for those with a body-mass index of less than 25.0 kg/m^2^ (Panel **G**). AUC denotes the area under the curve. The shaded area around each curve represents the 95% confidence interval.

The diagnostic performance of all four test protocols is shown in Table 3 and Figure 2. At the 30 min time point, the 45 mg test demonstrated excellent diagnostic accuracy, with an area under the receiver operating characteristic curve (AUC) of 0.953 (95% CI, 0.929 to 0.971). The 75 mg, 30 min protocol yielded a numerically higher AUC of 0.966 (95% CI, 0.945 to 0.981); however, this difference was not statistically significant (*p* = 0.50). Specifically, the 30 min sampling time yielded a significantly greater AUC compared with the 15 min time point for both the 45 mg dose (0.953 [95% CI, 0.929 to 0.971] vs. 0.906 [95% CI, 0.874 to 0.932]; *p* = 0.026) and the 75 mg dose (0.966 [95% CI, 0.945 to 0.981] vs. 0.925 [95% CI, 0.895 to 0.948]; *p* = 0.037). To comprehensively elucidate the temporal testing dynamics, we further evaluated the categorical agreement and continuous correlation between the two time points. For the 45 mg dose, the diagnostic agreement between the 15 and 30 min samples was 89.3% (Cohen’s kappa = 0.737), with a strong positive correlation in continuous delta-over-baseline values (Pearson’s r = 0.840; *p* < 0.001). Parallel findings were observed for the 75 mg dose, which demonstrated 91.2% diagnostic agreement (Cohen’s kappa = 0.791) and a robust linear correlation (Pearson’s r = 0.914; *p* < 0.001). Despite this substantial intra-dose concordance, the 30 min sampling protocol consistently provided superior diagnostic accuracy, establishing it as the optimal sampling threshold.

In key subgroup analyses, the comparable performance of the two 30 min protocols was consistent. Among participants younger than 40 years of age (*n* = 212), the AUC for the 45 mg protocol was 0.977, as compared with 0.943 for the 75 mg protocol, a difference that was not significant (*p* = 0.24) (Figure 2F). Similarly, in participants with a body-mass index of less than 25.0 kg/m^2^ (*n* = 334), the 45 mg protocol yielded an AUC of 0.966, as compared with 0.957 for the 75 mg protocol, a difference that was also not significant (*p* = 0.70) (Figure 2G).

To resolve the diagnostic discrepancies, the 42 participants with discordant UBT results at 30 min underwent the definitive endoscopic reference standard. Based on rapid urease testing and histology, 15 were confirmed as *H. pylori*-positive and 27 as *H. pylori*-negative. Among these 42 cases, the 75 mg protocol correctly identified significantly more *H. pylori*-positive participants than the 45 mg protocol (11 of 15 [73.3%] vs. 4 of 15 [26.7%]; *p* = 0.001). Conversely, the 45 mg protocol correctly identified significantly more *H. pylori*-negative participants (16 of 27 [59.3%] vs. 11 of 27 [40.7%]; *p* < 0.001) (Table 4).

### 3.4. Safety Outcomes

No adverse events of any grade were reported by any participant during the one-week follow-up period (Table 5).

### 3.5. Ancillary Analyses

In pre-specified subgroup analyses, the diagnostic agreement (as measured by Cohen’s kappa) between the 45 mg and 75 mg tests at 30 min was broadly consistent across most participant subgroups, including those defined by sex, alcohol use, smoking status, and family history of gastric cancer (Table 6). The agreement was substantial in both female participants (Cohen’s κ, 0.744) and male participants (κ, 0.676).

## 4. Discussion

In this prospective, randomized crossover trial, we demonstrated that a low-dose 45 mg ^13^C-UBT has substantial agreement and comparable diagnostic accuracy to the standard 75 mg test for the diagnosis of *H. pylori* infection in adults. Our findings support a more nuanced, potentially stratified, approach to test selection.

Our primary analysis revealed substantial agreement between the two protocols at the 30 min time point (κ, 0.766). This is consistent with previous research that has validated the efficacy of lower ^13^C-urea doses [18,33,34,35]. The consistency of this agreement across both randomization sequences confirms that the crossover design did not introduce significant bias. Furthermore, our confirmation that the 30 min sampling time is superior to the 15 min time point for both doses aligns with established guidelines, which emphasize adequate incubation for accurate results. This finding justifies focusing subsequent head-to-head comparisons on this optimal timing.

The central finding of our study was the statistical non-inferiority of the 45 mg protocol compared to the 75 mg standard at 30 min (AUC, 0.953 vs. 0.966; *p* = 0.50). While some earlier studies and international consensus reports have favored a 75 mg dose to maximize signal strength and sensitivity, our data contributes to a growing body of evidence suggesting that lower doses can achieve excellent diagnostic performance.

While our study focused on the 45 mg vs. 75 mg comparison, the quest for an “optimal” dose requires viewing our results within the broader evidence base. Prior studies have evaluated doses as low as 15–20 mg, often necessitating the addition of citric acid test meals to delay gastric emptying and enhance urease-substrate interaction. In our study, notably, the 45 mg tablet achieved excellent diagnostic accuracy without the inclusion of citric acid, a component present in the standard 75 mg granule formulation. This suggests that 45 mg represents an operational “sweet spot”: it provides sufficient substrate to yield a stable signal, statistically comparable to the acidified high-dose standard, while avoiding the complexity and palatability issues associated with citric acid, thus offering a simplified and patient-friendly low-dose option.

Our study’s most novel contribution, however, is the stratification by age and body-mass index. The robust performance of the 45 mg protocol in younger (<40 years) and lower-BMI (<25.0 kg/m^2^) individuals provides crucial new context. Physiologically, younger adults typically have higher gastric acid output and less atrophic gastritis compared to older populations, providing an optimal environment for urease activity. This supports the biological plausibility of using a lower 45 mg dose in this specific demographic. The lack of a significant difference compared to the 75 mg test in these large demographic groups strongly implies that the low-dose protocol is a highly effective tool for these individuals [36,37]. This has important practical implications, offering an opportunity to reduce medication dosage, lower costs, and minimize isotope exposure without compromising diagnostic yield, making it an attractive option for large-scale screening programs in younger populations.

Our analysis of discordant cases offers a nuanced perspective on the operational characteristics of each dose. The finding that the 75 mg protocol was more accurate for identifying true positive cases is in line with the rationale for its use in post-treatment testing, where maximizing sensitivity is critical [38,39]. Conversely, the superior accuracy of the 45 mg protocol in correctly identifying true negative cases suggests a higher specificity. This is consistent with systematic reviews proposing that lowering the urea dose may reduce the risk of false-positive results, possibly by minimizing urease activity from non-*H. pylori* urease-producing organisms. This suggests a potential strategy for tailored test selection: the 45 mg protocol may serve as an ideal, cost-effective tool for initial diagnosis or screening, while the 75 mg protocol remains valuable for high-stakes scenarios like confirming eradication. It is worth noting that our cohort included a proportion of patients undergoing post-eradication testing (approximately 22%). While the overall agreement between the two protocols remained high, future studies stratified strictly by clinical indication are warranted to evaluate if prior treatment alters the diagnostic dynamics of the low-dose formulation.

The safety profile of both protocols was excellent, with no adverse events reported. The diagnostic agreement was also found to be broadly consistent across other subgroups defined by sex, smoking, and alcohol use, further strengthening the generalizability of our findings [40]. This study has several strengths, including its prospective, randomized crossover design which minimizes inter-individual variability. Limitations include the single-center design, which may limit external validity. Second, while we reported the clinical indications for testing, we did not perform separate diagnostic accuracy analyses stratified by initial diagnosis versus post-eradication confirmation, nor did we grade the histologic severity of gastric inflammation. Third, detailed anthropometric data such as specific body weight and waist circumference were not collected, precluding a more granular correlation between patient body habitus and UBT values. Finally, Chinese patients typically have a lower mean stature and body weight compared to Western populations, which may influence gastric volume and isotope distribution. Therefore, while the 45 mg dose is highly effective in our cohort, these findings warrant reproduction in diverse ethnic populations.

In conclusion, the 45 mg, 30 min ^13^C-UBT provides substantial diagnostic agreement and comparable clinical performance to the 75 mg standard. While its robust performance in younger, lower-BMI individuals shows clinical promise, our diagnostic accuracy estimates rely partly on a composite reference assumption rather than independent endoscopic verification in all participants. Therefore, further externally validated studies using independent reference standards are required before broad screening-policy claims are made.

## Figures and Tables

**Table 1:** Baseline characteristics of the participants *.

Characteristic	Sequence A: 45 mg then 75 mg (*n* = 216)	Sequence B: 75 mg then 45 mg (*n* = 215)	*p*-Value
Age (years)	43.38 ± 15.62	40.62 ± 14.77	0.061
Sex (%)			0.224
Female	134 (62.04)	121 (56.28)	
Male	82 (37.96)	94 (43.72)	
BMI (kg/m^2^) ^†^	22.54 ± 3.24	22.64 ± 3.18	0.734
Smoking Status (%)			0.573
Current or Former	16 (7.41)	13 (6.05)	
Never	200 (92.59)	202 (93.95)	
Alcohol Consumption (%)			0.831
Current or Former	11 (5.09)	10 (4.65)	
Never	205 (94.91)	205 (95.35)	
Family History of Gastric Cancer (%)			0.993
Yes	7 (3.24)	7 (3.26)	
No	209 (96.76)	208 (96.74)	
Indication for testing			0.210
Initial diagnosis	163 (75.46)	173 (80.47)	
Post-eradication confirmation	53 (24.54)	42 (19.53)	

* Plus–minus values are means ± SD. Data for categorical variables are presented as number (percentage). There were no significant differences in baseline characteristics between the two randomized sequence groups. ^†^ BMI: The body-mass index is the weight in kilograms divided by the square of the height in meters.

**Table 2:** Diagnostic agreement between 45 mg and 75 mg ^13^C-urea breath test protocols *.

Characteristic	75 mg Test Result		Overall Agreement, % (No./Total No.)	Cohen’s Kappa (95% CI) ^‡^	McNemar *p* Value ^§^
	Positive	Negative			
30 min Time Point Comparison					
Total Population (*n* = 431)			90.3 (389/431)	0.766 (0.698 to 0.834)	0.088
45 mg Test, Positive	106	15			
45 mg Test, Negative	27	283			
Sequence A (*n* = 216)			90.3 (195/216)	0.776 (0.684 to 0.868)	0.189
45 mg Test, Positive	58	7			
45 mg Test, Negative	14	137			
Sequence B (*n* = 215)			90.2 (194/215)	0.754 (0.652 to 0.856)	0.383
45 mg Test, Positive	48	8			
45 mg Test, Negative	13	146			
15 min Time Point Comparison					
Total Population (*n* = 431)			87.0 (375/431)	0.686 (0.610 to 0.762)	0.504
45 mg Test, Positive	98	25			
45 mg Test, Negative	31	277			

* Data are for participants included in the per-protocol analysis. Sequence A: 45 mg test followed by 75 mg test; Sequence B: 75 mg test followed by 45 mg test. ^‡^ CI denotes confidence interval. Cohen’s kappa values are interpreted as follows: 0.61–0.80 as substantial and 0.81–1.00 as almost perfect agreement. ^§^ *p* value was calculated via the McNemar test to assess systematic disagreement between the paired tests.

**Table 3:** Diagnostic performance of ^13^C-urea breath test protocols at optimal cutoffs in the per-protocol population *.

Test Protocol and Time Point	Optimal Cutoff ^†^ (δ/L)	Sensitivity, % (95% CI)	Specificity, % (95% CI)	PPV, % (95% CI)	NPV, % (95% CI)	Youden’s Index (J)	Area Under ROC Curve (95% CI)
45 mg Dose							
15 min Sample	>3.9	86.1 (78.6 to 91.7)	89.6 (85.7 to 92.8)	77.2 (69.4 to 83.8)	93.9 (90.5 to 96.3)	0.757	0.906 (0.874 to 0.932)
30 min Sample	>3.9	92.6 (86.5 to 96.6)	94.2 (90.9 to 96.5)	86.3 (79.5 to 91.5)	97.0 (94.4 to 98.6)	0.868	0.953 (0.929 to 0.971) ^‡^
75 mg Dose							
15 min Sample	>5.1	86.1 (78.6 to 91.7)	92.9 (89.4 to 95.5)	82.7 (75.2 to 88.6)	94.4 (91.3 to 96.6)	0.790	0.925 (0.895 to 0.948) ^§¶^

* The diagnostic performance was evaluated against the composite reference standard(concordant UBT results presumed as true status; discordant cases verified by endoscopy). Analyses were based on the per-protocol population with a definitive *H. pylori* status (*n* = 431), which included 122 positive and 309 negative cases. CI, confidence interval; NPV, negative predictive value; PPV, positive predictive value; ROC, receiver-operating characteristic. Confidence intervals were calculated with the use of the Wilson score method. ^†^ The optimal cutoff value for the delta-over-baseline (DOB) value was determined by maximizing the Youden index (J). ^‡^ *p* = 0.03 for the comparison with the 15 min sample in the 45 mg dose group. ^§^ *p* = 0.04 for the comparison with the 15 min sample in the 75 mg dose group. ^¶^ *p* = 0.50 for the comparison with the 45 mg, 30 min test. All *p* values for AUC comparisons were calculated with the use of the DeLong method.

**Table 4:** Analysis of discordant results between the 45 mg and 75 mg protocols at 30 min *.

Reference Standard Status	No. of Discordant Cases	Correct Classifications by 45 mg Protocol, No. (%)	Correct Classifications by 75 mg Protocol, No. (%)	*p* Value ^†^
Positive for *H. pylori*	15	4 (26.7)	11 (73.3)	0.001
Negative for *H. pylori*	27	16 (59.3)	11 (40.7)	<0.001

* Data are for the 42 participants with discordant results between the 45 mg and 75 mg protocols at the 30 min time point. A correct classification for a positive case is a true positive; a correct classification for a negative case is a true negative. ^†^ *p* values were calculated with the use of Fisher’s exact test to compare the proportions of correct classifications between the two protocols within each stratum of the reference standard status.

**Table 5:** Adverse events in the safety population *.

Event Category	Sequence A (45 mg → 75 mg) (*n* = 216)	Sequence B (75 mg → 45 mg) (*n* = 215)	Total (*n* = 432)
Any adverse event—no. of participants (%)	0 (0.0)	0 (0.0)	0 (0.0)
Serious adverse event—no. (%)	0 (0.0)	0 (0.0)	0 (0.0)
Adverse event leading to withdrawal—no. (%)	0 (0.0)	0 (0.0)	0 (0.0)

* The safety population included all 432 randomized participants. Data are presented as the number of participants with an event (percentage). No adverse events of any grade were reported by any participant during the one-week follow-up period.

**Table 6:** Subgroup analyses of diagnostic agreement between 45 mg and 75 mg tests at 30 min *.

Subgroup	Category	No. of Participants	Cohen’s Kappa (95% CI) ^†^
Overall	Total Population	431	0.766 (0.700 to 0.832)
Age Group	<40 years	212	0.689 (0.578 to 0.799)
	≥40 years	219	0.832 (0.753 to 0.911)
Sex	Female	255	0.744 (0.648 to 0.840)
	Male	176	0.676 (0.564 to 0.788)
Body-Mass Index	<25.0 kg/m^2^	334	0.786 (0.713 to 0.860)
	≥25.0 kg/m^2^	97	0.699 (0.548 to 0.850)
Smoking Status	Never	402	0.785 (0.718 to 0.852)
	Current or former	29	0.494 (0.161 to 0.827)
Alcohol Use	Never	410	0.769 (0.700 to 0.838)
	Current or former	21	0.704 (0.396 to 1.000)
Family History of Gastric Cancer	No	417	0.760 (0.691 to 0.829)
	Yes	14	1.000 ^‡^

* Analyses were based on the per-protocol population, comparing the results of the 45 mg and 75 mg ^13^C-urea breath tests at the 30 min time point. ^†^ CI denotes confidence interval. Confidence intervals were calculated using the standard error from the statistical output (CI = κ ± 1.96 × SE). ^‡^ The kappa coefficient is based on a very small number of participants and should be interpreted with caution.

## Data Availability

The data that support the findings of this study are available from the corresponding author upon reasonable request.

## Supplementary Materials

The following supporting information can be downloaded at https://www.mdpi.com/article/10.3390/diagnostics16101567/s1: File S1 CONSORT 2025 checklist.
