# Correction: Huang et al. Accuracy and Agreement of a 45 mg Versus a 75 mg ^13^C-Urea Breath Test for the Diagnosis of *Helicobacter pylori* Infection in Adults: A Randomized Crossover Trial. *Diagnostics* 2026, *16*, 1567

**DOI:** 10.3390/diagnostics16193071

**Published:** 2026-09-22

**Authors:** Jun Huang, Yaohong Xie, Jian Huang, Bingyun Lu, Ye Chen

**Affiliations:** 1 Guangdong Provincial Key Laboratory of Gastroenterology, Department of Gastroenterology, Nanfang Hospital, Southern Medical University, Guangzhou 510000, China; wlncsft@smu.edu.cn (J.H.); yaohongxie2023@163.com (Y.X.); 2 Integrative Microecology Clinical Center, Shenzhen Key Laboratory of Gastrointestinal Microbiota and Disease, Shenzhen Clinical Research Center for Digestive Disease, Shenzhen Technology Research Center of Gut Microbiota Transplantation, Department of Gastroenterology, Shenzhen Hospital, Southern Medical University, Shenzhen 518000, China; 18385336215@163.com

There was an error in the original publication [1]. The clinical trial was incorrectly stated as prospectively registered, and the required details for a retrospective registration were missing.

A correction has been made to Section 2.1. Trial Design and Oversight, Paragraph 2:

This randomized crossover trial was retrospectively registered at the Chinese Clinical Trial Registry, registration number ChiCTR2400090596, on 9 October 2024, after the start of patient enrollment on 10 October 2023. The delay in registration resulted from an administrative oversight. The authors confirm that ethics approval and informed consent were obtained before enrollment and that the registered outcomes are consistent with those reported in this article.

The authors state that the scientific conclusions are unaffected. This correction was approved by the Academic Editor. The original publication has also been updated.
